# Inaccuracy of intraocular pressure measurement in congenital corneal opacity: three case reports

**DOI:** 10.1186/s12886-019-1287-7

**Published:** 2020-01-02

**Authors:** Byeong Soo Kang, Jin Wook Jeoung, Joo Youn Oh

**Affiliations:** 1 Department of Ophthalmology, Seoul National University College of Medicine, 103, Daehak-ro, Jongno-gu, Seoul, 03080 South Korea; 2 Laboratory of Ocular Regenerative Medicine and Immunology, Biomedical Research Institute, Seoul National University Hospital, 101, Daehak-ro, Jongno-gu, Seoul, 03080 South Korea

**Keywords:** Applanation-based tonometry, Case report, Congenital corneal opacity, Congenital glaucoma, Intraocular pressure

## Abstract

**Background:**

To report three cases of congenital corneal opacity where intraocular pressure (IOP) readings were high despite the use of multiple anti-glaucoma eye drops and normalized after corneal transplantation.

**Case presentation:**

Three Korean infants presented with bilateral dense stromal opacification which had been present since birth. IOPs measured by rebound tonometer were high despite administration of multiple anti-glaucoma medications. One eye of each patient underwent penetrating keratoplasty (PK) because corneal opacity impaired visual development. Immediately after PK, IOPs were normalized and maintained normal without medication, whereas they remained high in the contralateral unoperated eye. On histology, stromal fibrosis was observed in the removed corneal button, and molecular assays revealed increased levels of type 1 and 5 collagens.

**Conclusion:**

The IOP measurement using the conventional applanation-based tonometry can be inaccurate in congenital corneal opacity which is marked by corneal fibrosis. Therefore, IOP values should be interpreted with caution in these patients, and the possibility of false-positive diagnosis of glaucoma considered.

## Background

Congenital corneal opacity and congenital glaucoma commonly coexist in pediatric patients with anterior segment dysgenesis. The diagnosis of glaucoma in these patients is difficult because corneal opacification obscures visualization of intraocular structures such as angle and optic disc. Moreover, the measurement of intraocular pressure (IOP) using conventional applanation-based tonometry is inaccurate in the abnormal cornea [1].

Herein, we report three Korean pediatric patients with congenital corneal opacity presenting as dense stromal opacification. In the patients, IOP readings were high despite the use of multiple anti-glaucoma eye drops. Immediately after corneal transplantation, IOPs were normalized and maintained within a normal range in the absence of anti-glaucoma medication. Histologic and molecular analysis showed high levels of collagen deposition in the corneal stroma.

## Case presentation

The study was approved by the Institutional Review Board of Seoul National University Hospital.

### Case 1

**Fig. 1:**
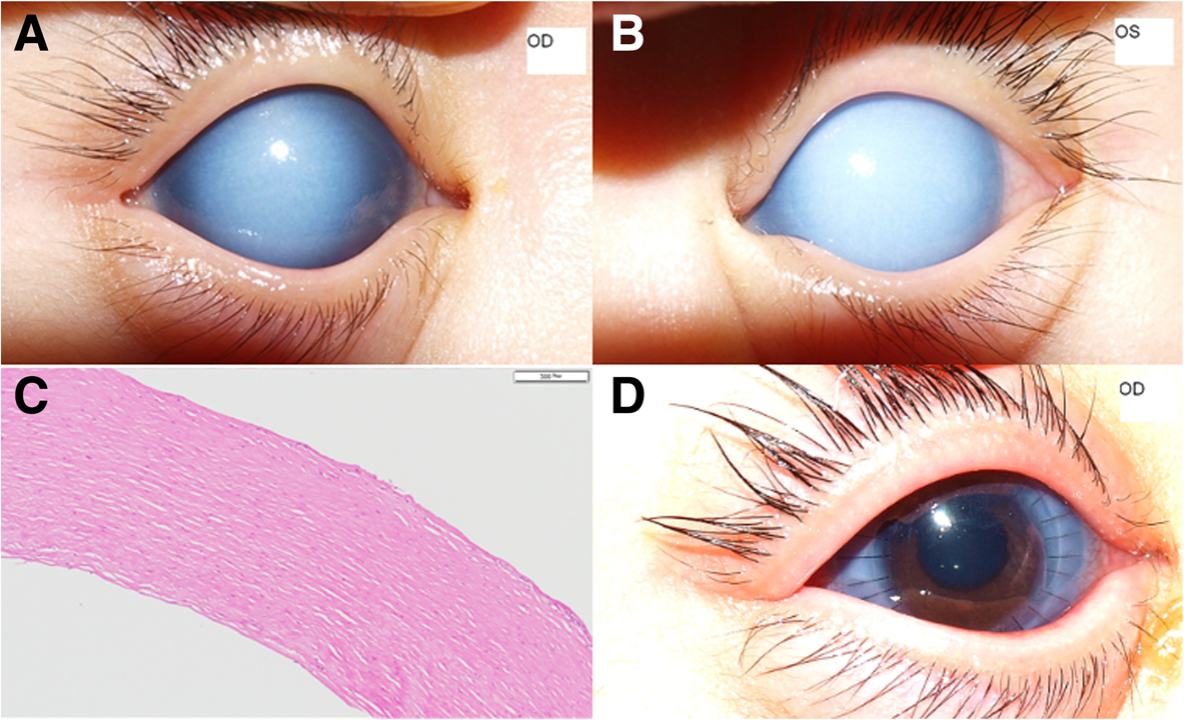
Case 1. **a**, **b** Anterior segment photography of the right (**a**) and left (**b**) eyes taken at the initial visit to our clinic (7 months after birth). The intraocular pressure (IOP) values measured by a rebound tonometer were 42.5 mmHg OD and 45.1 mmHg OS. **c** Hematoxylin-eosin staining of the right cornea excised at the time of penetrating keratoplasty (PK). Scale bar, 200 μm. **d** Anterior segment photograph of the right eye 1 day after PK. The IOP by a rebound tonometer was 18.8 mmHg OD

A 7-month-old girl was referred to our clinic for corneal opacity and glaucoma that had been present in both eyes since birth. She was born at full-term weighing 2.7 kg and received a brief intensive care unit care due to tachypnea. Otherwise, the infant was systemically healthy, and all serological test results for TORCH (toxoplasmosis, rubella, cytomegalovirus, and herpes simplex virus) embryopathy were negative. Slit lamp examination showed dense stromal opacity in the central cornea in both eyes (Fig. 1a, b). The corneal diameter was 11 mm × 10.5 mm in both eyes, and ultrasound pachymetry failed. Anterior chamber was formed, and partial aniridia was observed. The fundus was invisible, but ultrasonography showed no abnormality in the posterior segment. IOP was 42.5 mmHg in the right eye and 45.1 mmHg in the left as measured by a rebound tonometer (Icare® PRO; Icare, Helsinki, Finland). Despite combined treatment of 0.005% latanoprost (Xalatan®; Pfizer, New York, NY) QD and 2% dorzolamide/0.5% timolol (Cosopt®; MSD, Riom, France) BID, the IOP did not decrease in both eyes. To correct corneal opacity, penetrating keratoplasty (PK) was performed in the right eye. Histologic examination of an excised corneal button revealed stromal fibrosis (Fig. 1c). One day after PK, the IOP was reduced to 18.8 mmHg in the right eye without administration of any IOP-reducing agents (Fig. 1d), while it remained high in the other eye. The IOP in the right eye was maintained within normal range for 36 months of follow-up without the use of anti-glaucoma medication.

### Case 2

**Fig. 2:**
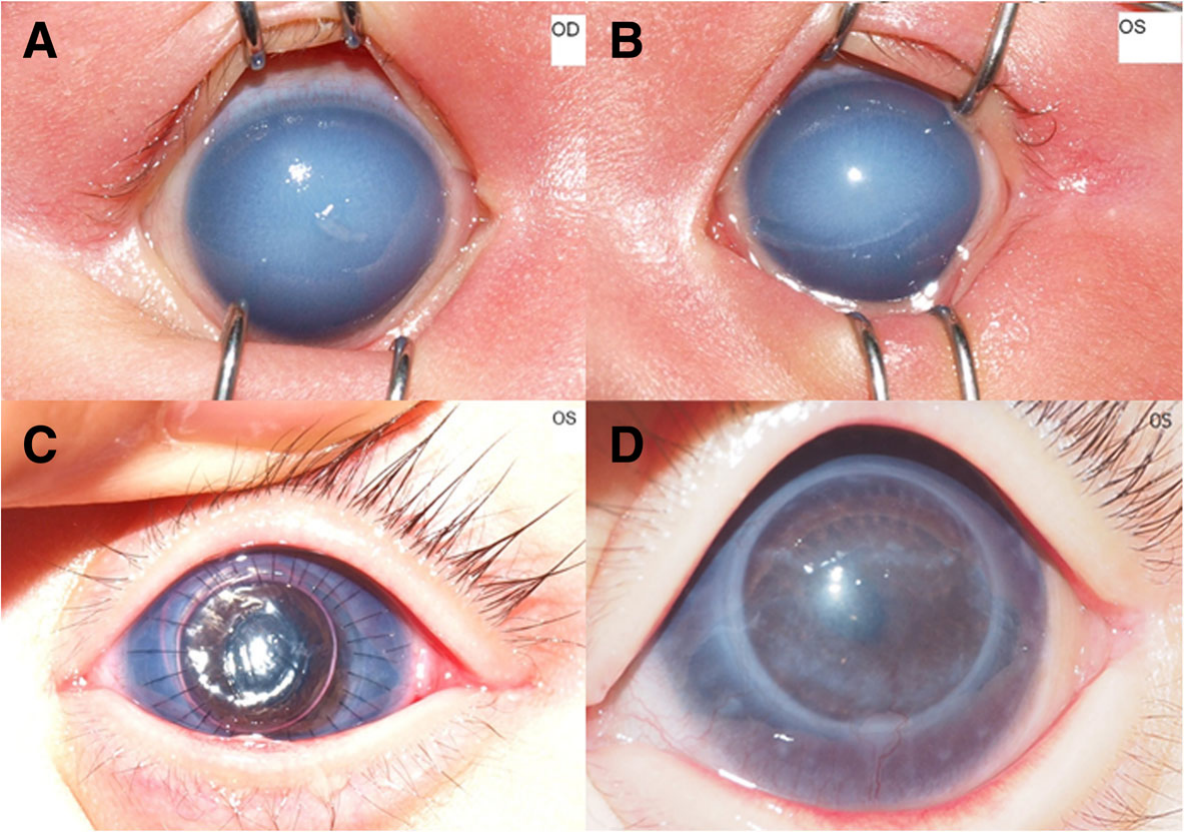
Case 2. **a**, **b** Anterior segment photography of the right (**a**) and left eyes (**b**) 15 days after birth. The intraocular pressure (IOP) was 32.5 mmHg OD and 26.4 mmHg OS by rebound tonometer. **c** Anterior segment photograph of the left eye 2 days after penetrating keratoplasty (PK). IOP was reduced to 21.0 mmHg OS. **d** Anterior segment photograph of the left eye 18 months after surgery. The IOP was 17.5 mmHg without anti-glaucoma medication OS

A 15-day-old boy visited our clinic because of bilateral corneal opacity present at birth. He was born full-term at 3.24 kg. At initial examination, dense central corneal opacity was observed in both eyes (Fig. 2a, b). The anterior chamber was present, and iris was normal. No specific findings were seen in the retina or vitreous cavity on ultrasonography. The corneal diameter was 12 mm × 11.5 mm in the right eye and 11 mm × 10.5 mm in the left. The corneal thickness was unmeasurable by ultrasonic pachymeter. The IOP was 32.5 mmHg in the right eye and 26.4 mmHg in the left eye by Icare® rebound tonometer. The patient started instillation of 0.005% latanoprost (Xalatan®) QD and 1% brinzolamide/0.5% timolol (Elazop®, Alcon, Fort Worth, TX) BID, and was referred to a pediatrician for examination of associated systemic anomalies. No systemic abnormality was detected, and the TORCH screen results were negative. Despite anti-glaucoma medications, IOP remained high in both eyes, and corneal opacity was severe enough to impair visual development. Hence, PK was performed in the left eye at the age of 4 months. In the operation room, IOP was 29.4 mmHg in the right eye and 30.5 mmHg in the left even after systemic administration of mannitol and topical anti-glaucoma eye drop instillation. Two days after surgery, the corneal graft was clear (Fig. 2c), and IOP dropped to 21.0 mmHg in the left eye in the absence of any IOP-lowering treatment. At last follow-up (post-operative 36 months), the graft was clear (Fig. 2d), and IOP was 17.5 mmHg without using anti-glaucoma eye drops. However, IOP in the unoperated eye remained high (32.8 mmHg) even with Xalatan® QD and Cosopt® BID.

We additionally analyzed the excised cornea for levels of collagen type I, V, and lumican by real-time RT PCR and compared with control corneas obtained from two patients who underwent PK due to congenital hereditary endothelial dystrophy (CHED). Results demonstrated that the mRNA levels of type I and V collagens were 19.7 and 7.5 times higher in the patient than in the control (Table 1).

**Table 1:** Real time RT-PCR analysis of the cornea for extracellular matrix proteins

	Collage type 1	Collagen type 5	Luimcan
Case 2	19.7^a^	7.5	1.19
Case 3	9.5	2.8	0.39
Control 1^b^	1	1	1
Control 2^b^	1.25	0.96	0.10

^a^Data are presented as a fold change relative to control corneas

^b^Control corneas were obtained from patients with CHED at the time of penetrating keratoplasty (PK). PK was performed at 4 years of age in control 1 and 10 months in control 2

### Case 3

**Fig. 3:**
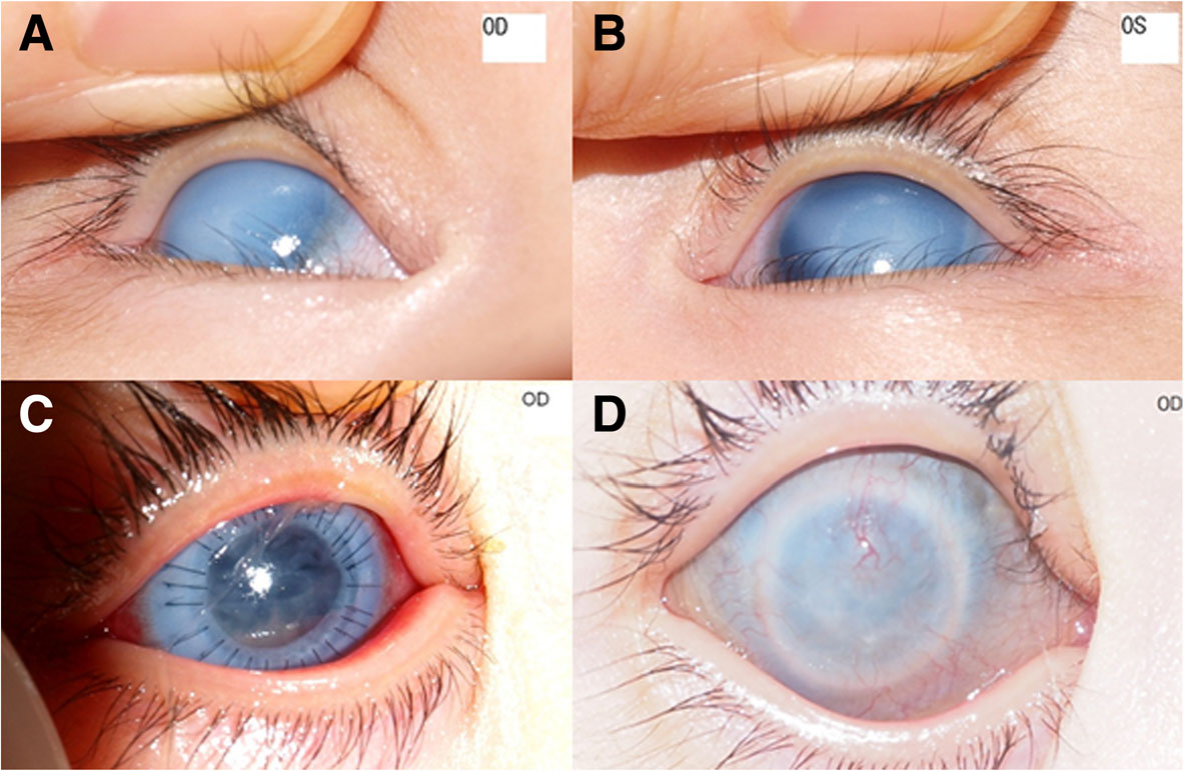
Case 3. **a**, **b** Anterior segment photography of the right (**a**) and left eyes (**b**) 3 months after birth. The intraocular pressure (IOP) values were 38.1 mmHg OD and 35.7 mmHg OS as measured by rebound tonometer. **c** Anterior segment photograph of the right eye 1 day after penetrating keratoplasty (PK). The IOP was 14.2 mmHg OD. **d** Anterior segment photograph of the right eye at the post-operative 4 months. The IOP by a rebound tonometer was 21.3 mmHg OD without medication

A 3-month-old girl was referred to our clinic for congenital corneal opacity and increased IOP in both eyes. According to the medical report, she was a full-term baby and systemically healthy except for corneal opacity at birth. Her IOPs were 32.0 and 31.0 mmHg in the right and left eyes, respectively when measured 1 day after birth, and therefore, she started treatment with Xalatan® QD and Elazop® BID since 4 days after birth. TORCH work-up revealed negative results. At first examination in our clinic, total corneal opacity and complete aniridia were found in both eyes (Fig. 3a, b). The corneal diameter was 11 mm × 10.5 mm and thickness uncheckable. IOP was 38.1 mmHg in the right eye and 35.7 mmHg in the left eye by a rebound tonometer (Icare® PRO). At 10 months of age, the patient underwent PK in the right eye. Prior to surgery, mannitol was intravenously administered and Xalatan® and Elazop® eye drops instilled, but the IOP was checked 31.5 mmHg. During surgery, the lens was spontaneously delivered right after trephination of the recipient cornea, and therefore, lens removal was performed together with corneal transplantation. On the next day, the IOP was reduced to 14.2 mmHg without anti-glaucoma treatment (Fig. 3c), and the anti-glaucoma medication was discontinued in the right eye thereafter. Four months of surgery, the graft rejection occurred, and the cornea was opacified with vascularization (Fig. 3d). Nonetheless, the IOP in the right eye was maintained within a range of normal values without medication for 34 months of follow-up, while it remained high (42.3 mmHg) in the left eye even with medication.

Real-time RT PCR analysis of the excised cornea at the time of surgery revealed that the mRNA levels of collagen type I and V were 9.5 and 2.8 times higher, respectively, compared to those in control corneas (Table 1).

## Discussion and conclusions

The conventional methods for IOP measurement are based on corneal applanation and measure the force required to flatten a constant area of the cornea. Therefore, IOP measurement by applanation-based tonometry largely depends on biomechanical properties of the cornea, and tonometry artefacts can occur in abnormal corneas [1]. For example, IOP reading can be artificially high in the eye with high central corneal thickness [2]. Keratoconus, prior keratorefractive surgery, or corneal cross-linking procedure can alter corneal hysteresis (CH), leading to IOP mismeasurement [3–6]. Also, CH can be affected by corneal fibrosis. For example, corneal opacification occurred as a result of cicatrization and fibrotic changes in the anterior stroma in patients with mucopolysaccharidosis (MPS) [7], and CH was reported to be higher in MPS patients as assessed by Ocular Response Analyzer [8]. High CH values overestimate IOP [9]. Therefore, it is possible that corneal stromal fibrosis and the resultant high CH lead to higher IOP readings by applanation tonometer than true IOP values.

The rebound tonometer, a recently developed hand-held tonometer, is considered safe and suitable for a pediatric population, because corneal application by a rebound tonometer is fast and corneal anesthesia is not required. In addition, it was reported that IOP values measured by rebound tonometer in pediatric glaucoma patients were similar to those by Goldmann applanation tonometer [10, 11]. However, IOP measurements by rebound tonometer can be affected by central corneal thickness and corneal biomechanical properties [12–14].

Despite high IOP readings as measured by rebound tonometer (upto or over 30 mmHg), the corneal size was normal (≤ 11 mm of diameter) and there were no symptoms of epiphora, photophobia and blepharospasm (the classical triad of symptoms in congenital glaucoma) in our patients. Moreover, administration of multiple anti-glaucoma medications was not effective in reducing IOPs. For these reasons, we suspected that the increased IOP measurements in our patients might not be due to congenital glaucoma but derived from thickened and stiffened corneas, and performed PK to clear the visual axis from dense opacity. Consequently, IOPs were immediately normalized by corneal transplantation and thereafter controlled without medication, whereas the IOPs in the unoperated eye remained high with using anti-glaucoma medication. The corneal opacity in our cases was clinically dense central stromal opacification in both eyes. Histologically, stromal fibrosis was observed (case 1), and molecular assay revealed increased levels of type 1 and 5 collagens which are the main extracellular matrix components of the corneal stroma (cases 2 and 3). Although we did not directly measure CH in these patients, it is likely that corneal fibrosis might increase corneal resistance to applanation, thereby leading to overestimation of IOPs by rebound tonometry.

In summary, our cases demonstrate that the conventional IOP measurement method using transcorneal approach might be incorrect and inadequate in congenital corneal opacity where CH is altered due to corneal fibrosis. In these patients, IOP values should be interpreted with caution, and the possibility of false-positive diagnosis of glaucoma considered. Eventually, new technology for measuring IOP independently of corneal biomechanical properties is needed for accurate IOP measurement and glaucoma diagnosis.

## Data Availability

All the data are presented in the main paper.

## Abbreviations

CH: Corneal hysteresis
CHED: Congenital hereditary endothelial dystrophy
IOP: Intraocular pressure
MPS: Mucopolysaccharidosis
PCR: Polymerase chain reaction
PK: Penetrating keratoplasty
TORCH: Toxoplasmosis, rubella, cytomegalovirus, and herpes simplex virus
